# Modifiable Risk Factor Score and Fecundability in a Preconception Cohort in Singapore

**DOI:** 10.1001/jamanetworkopen.2022.55001

**Published:** 2023-02-07

**Authors:** See Ling Loy, Chee Wai Ku, Michelle Mei Ying Tiong, Carissa Shi Tong Ng, Yin Bun Cheung, Keith M. Godfrey, Shan Xuan Lim, Marjorelee T. Colega, Jun Shi Lai, Yap-Seng Chong, Lynette Pei-Chi Shek, Kok Hian Tan, Shiao-Yng Chan, Mary Foong-Fong Chong, Fabian Yap, Jerry Kok Yen Chan

**Affiliations:** 1 Department of Reproductive Medicine, KK Women’s and Children’s Hospital, Singapore, Singapore; 2 Duke-NUS Medical School, Singapore, Singapore; 3 KK Women’s and Children’s Hospital, Singapore, Singapore; 4 Yong Loo Lin School of Medicine, National University of Singapore, National University Health System, Singapore, Singapore; 5 Program in Health Services and Systems Research and Center for Quantitative Medicine, Duke-NUS Medical School, Singapore, Singapore; 6 Tampere Center for Child, Adolescent and Maternal Health Research, Tampere University, Tampere, Finland; 7 Medical Research Council Lifecourse Epidemiology Centre, University of Southampton, Southampton, United Kingdom; 8 National Institute for Health Research Southampton Biomedical Research Centre, University of Southampton and University Hospital Southampton National Health Service Foundation Trust, Southampton, United Kingdom; 9 Saw Swee Hock School of Public Health, National University of Singapore, National University Health System, Singapore, Singapore; 10 Singapore Institute for Clinical Sciences, Agency for Science, Technology and Research, Singapore, Singapore; 11 Department of Paediatrics, Yong Loo Lin School of Medicine, National University of Singapore, National University Health System, Singapore, Singapore; 12 Khoo Teck Puat-National University Children’s Medical Institute, National University Hospital, National University Health System, Singapore, Singapore; 13 Department of Maternal Fetal Medicine, KK Women’s and Children’s Hospital, Singapore, Singapore; 14 Department of Obstetrics and Gynaecology, Yong Loo Lin School of Medicine, National University of Singapore, National University Health System, Singapore, Singapore; 15 Department of Paediatrics, KK Women’s and Children’s Hospital, Singapore, Singapore; 16 Lee Kong Chian School of Medicine, Nanyang Technological University, Singapore, Singapore

## Abstract

**Importance:**

Although multiple modifiable risk factors have been identified for reduced fecundability (defined as lower probability of conception within a menstrual cycle), no scoring system has been established to systematically evaluate fecundability among females who are attempting to conceive.

**Objective:**

To examine the association of a risk score based on 6 modifiable factors with fecundability, and to estimate the percentage reduction in incidence of nonconception if all study participants achieved a minimal risk score level.

**Design, Setting, and Participants:**

This population-based cohort study obtained data from the S-PRESTO (Singapore Preconception Study of Long-Term Maternal and Child Outcomes) prospective cohort study. Females of reproductive age who were trying to conceive were enrolled from February 2015 to October 2017 and followed for 1 year, ending in November 2018. Data were analyzed from March to May 2022.

**Exposures:**

A reduced fecundability risk score was derived by giving participants 1 point for each of the following factors: unhealthy body mass index, unhealthy diet, smoking, alcohol intake, folic acid supplement nonuser, and older maternal age. Total scores ranged from 0 to 6 and were classified into 5 levels: level 1 (score of 0 or 1), level 2 (score of 2), level 3 (score of 3), level 4 (score of 4), and level 5 (score of 5 or 6).

**Main Outcomes and Measures:**

Fecundability, measured by time to conception in cycles, was analyzed using discrete-time proportional hazards models with confounder adjustment.

**Results:**

A total of 937 females (mean [SD] age, 30.8 [3.8] years) were included, among whom 401 (42.8%) spontaneously conceived within 1 year of attempting conception; the median (IQR) number of cycles before conception was 4 (2-7). Compared with participants with a level 1 risk score, those with level 2, 3, 4, and 5 risk scores had reductions in fecundability of 31% (adjusted fecundability ratio [FR], 0.69; 95% CI, 0.54-0.88), 41% (FR, 0.59; 95% CI, 0.45-0.78), 54% (FR, 0.46; 95% CI, 0.31-0.69) and 77% (FR, 0.23; 95% CI, 0.07-0.73), respectively. Assessment of the population attributable fraction showed that all participants achieving a minimal (level 1) risk level would be associated with a reduction of 34% (95% CI, 30%-39%) in nonconception within a year.

**Conclusions and Relevance:**

Results of this study revealed the co-occurrence of multiple modifiable risk factors for lowered fecundability and a substantially higher conception rate among participants with no or minimal risk factors. The risk assessment scoring system proposed is a simple and potentially useful public health tool for mitigating risks and guiding those who are trying to conceive.

## Key Points

**Question:**

Can a risk scoring system that is based on modifiable risk factors, such as unhealthy body mass index, unhealthy diet, smoking status, alcohol intake, nonuse of folic acid supplements, and older age, be used to systematically assess fecundability in females?

**Findings:**

In this cohort study of 937 females of reproductive age in Singapore who were trying to conceive over a 1-year period, a risk factor scoring tool assessing modifiable risk factors was proposed and revealed that a higher risk score level was associated with lower fecundability.

**Meaning:**

Findings of this study suggest that the risk scoring system proposed is a simple and potentially useful public health tool for highlighting the factors associated with reduced fecundability and for guiding individuals and couples in mitigating these risks.

## Teaser

This cohort study assesses the association between lifestyle factors and probability of conception within a menstrual cycle among females of reproductive age in Singapore.

## Introduction

Fecundability, measured by time to conception, is defined as the probability of conception in a month or in a menstrual cycle.^1^ Due to decreasing fertility rates globally,^2,3^ risk factors associated with fecundability are of interest to clinicians and couples trying to conceive, since 10% to 16% of couples in the US have difficulty conceiving.^4,5^ Especially important are lifestyle risk factors for reduced fecundability that are potentially modifiable, such as unhealthy weight, unhealthy diet, smoking, and alcohol intake.^6^ Although these risk factors have been widely studied, there is no established risk scoring system that integrates these lifestyle factors to guide individuals, especially those in the community, on how to improve their chances of natural conception to lessen the need to seek treatment in fertility clinics. In addition, there is no fecundability screening tool to facilitate patient risk stratification during the preconception period.

Using data from the Singapore Preconception Study of Long-Term Maternal and Child Outcomes (S-PRESTO) cohort, we examined the association of a risk score based on 6 modifiable factors with fecundability among females who were trying to conceive, and we estimated the percentage reduction in incidence of nonconception if all individuals achieved a minimal risk score level. Such a holistic risk score could be used as a public health and clinical assessment tool to evaluate risk factors for reduced fecundability in individuals of reproductive age, guiding improvement in fertility and shortening time to conception.

## Methods

### Study Participants and Procedures

From February 2015 to October 2017, females from the general population in Singapore who were attempting to conceive were recruited to participate in the S-PRESTO prospective cohort study.^7^ Recruitment involved social media advertisements, community leaflet distributions, and invitation letters mailed to female patients of reproductive age who were registered at KK Women’s and Children’s Hospital in the past 5 years and were currently not receiving active treatment. Inclusion criteria were 18 to 45 years of age; Chinese, Indian, or Malay ethnicity (self-identified); and attempting conception within the next 12 months. Exclusion criteria included known type 1 or 2 diabetes, use of anticonvulsant medication or oral corticosteroids, and assisted fertility treatment in the past month. The present population-based cohort study was conducted according to the guidelines in the Declaration of Helsinki.^8^ Ethics approval was obtained from the SingHealth Centralised Institutional Review Board. All participants provided written informed consent. We followed the Strengthening the Reporting of Observational Studies in Epidemiology (STROBE) reporting guideline.^9^

At baseline, participants completed interviewer-administered questionnaires about sociodemographic factors, health history, reproductive factors, and lifestyle habits as well as a validated Food Frequency Questionnaire.^10^ Trained research staff performed anthropometric measurements. Participants were instructed to perform a pregnancy test at home, using a urine pregnancy test kit (Biotron Diagnostics) we provided, if their menstrual period was 3 to 4 days late or 2 weeks after unprotected intercourse. An ultrasonography scan was scheduled after notification of a positive pregnancy test. Participants were contacted after 3, 6, and 12 months of recruitment to verify their conception status if no prior notification had been received. Follow-up ended in November 2018.

### Assessment of Modifiable Risk Factors

Weight was measured with a digital scale (Seca 803), and height was measured with a portable stadiometer (Seca 213; Seca). Body mass index (BMI) was calculated as weight in kilograms divided by height in meters squared. Using thresholds for Asian populations,^11^ we classified a BMI lower than 18.5 (underweight) or 23 or higher (overweight or obese) as unhealthy, while a BMI of 18.5 to 22.9 was classified as healthy.

A validated semiquantitative 92-item Food Frequency Questionnaire was used to ascertain habitual dietary intake in the past month.^10^ Participants reported the frequency of food and beverage intake, followed by the respective portion size. These values were converted to obtain daily intake in serving size. To facilitate the application of the risk score in populations, we used the vegetable component as a surrogate marker for a healthful diet, based on evidence showing that a healthy plant-based diet was associated with increased fecundability.^12^ Vegetable intake comprising a sum of leafy vegetables, cruciferous vegetables, and peas was classified into less than 2 servings per day to represent an unhealthy diet or 2 or more servings per day to represent a healthy diet.^13^

Participants were asked whether they had ever smoked regularly (at least once a day for a year or more) and were currently smoking, and their status was then classified as ever (current or past) smokers or never smokers. Participants were also asked about their alcohol drinking habits in the past 3 months, including the frequency and standard amount of alcohol intake by type of drinks. The total number of drinks was calculated and classified as 1 or fewer standard drink per week or more than 1 standard drink per week.^14,15^ Participants were asked if they had consumed any pills, tonics, or tablets to supplement their diet in the past 3 months; we identified and classified those with supplements containing folic acid (single or multivitamin/mineral form) as users or nonusers of folic acid supplements.^16,17^ Age at recruitment was classified as younger than 32 years or 32 years or older, based on evidence that female fertility decreases substantially starting approximately at age 32 years.^18^

### Definition of Risk Score

The risk score was generated based on previous evidence from the S-PRESTO cohort^12,17,19^ and a priori published risk factors for reduced fecundability.^1,14,15,16,20,21^ Participants received a 1-point score for each of the following: unhealthy BMI (<18.5 or ≥23), unhealthy diet (vegetable intake <2 servings per day), ever smoker status, alcohol intake of more than 1 standard drink per week, folic acid supplement nonuser, and 32 years or older. By summing the scores for each of these 6 factors, the risk score ranged from 0 (most healthy and youngest) to 6 (least healthy and oldest). Since only 30 participants scored 0, we grouped together those with scores of 0 or 1. Similarly, few participants scored 5 (n = 22) or 6 (n = 1), and thus they were also combined into 1 group. Five levels of risk score were derived: level 1 (score of 0 to 1), level 2 (score of 2), level 3 (score of 3), level 4 (score of 4), and level 5 (score of 5 to 6).

### Assessment of Conception and Cycles at Risk

The event of interest was conception according to a positive urine pregnancy test result, as confirmed by the presence of an intrauterine gestational sac on ultrasonography after 6 weeks of amenorrhea.^22,23^ We ascertained time to conception based on the number of menstrual cycles required to achieve conception within 1 year of follow-up. This number was calculated from the interval between the last menstrual period dates at baseline and before conception (for those who were pregnant) or last follow-up call (for those who were censored), divided by the mean menstrual cycle length. Overall, participants contributed observed cycles at risk from study entry until the occurrence of 1 of the following events: conception, initiation of fertility treatment, loss to follow-up, or 1 year after recruitment, whichever occurred first. The total number of cycles at risk was estimated as previously described.^22,23^

### Statistical Analysis

We used discrete-time proportional hazards models, with time to conception in menstrual cycles (discrete scale) as the underlying time axis to estimate fecundability ratios (FRs) and 95% CIs, the mean per cycle probability of conception in exposed vs unexposed participants.^24,25^ An FR higher than 1 indicates increased fecundability with shorter time to conception, and an FR lower than 1 indicates reduced fecundability with longer time to conception. The models accounted for left truncation and right censoring.^22,23^ We adjusted the models for variables that could be potential confounders, including ethnicity, educational level, monthly household income, and parity. These variables were identified from the literature^26,27,28^ and based on a directed acyclic graph. Risk score level was analyzed as a categorical or continuous variable.

We tested the interactions of risk score with ethnicity, educational level, monthly household income, and parity on fecundability by introducing each of the cross-product terms (eg, risk score by parity) into the fully adjusted models. No stratification analyses were performed given that all *P* for interaction >.15. We constructed curves of the cumulative percentage of participants who successfully conceived or delivered live births by risk score levels, which were estimated using the Kaplan-Meier method. We performed additional analysis to estimate fecundability by treating the event of interest as a live birth pregnancy outcome (n = 342).

In secondary analyses, instead of vegetable intake of less than 2 servings per day, we used a different definition of unhealthy diet as a risk factor: the median level of healthful plant-based diet index (PDI) with a score of 47 or lower in pregnant participants.^12^ We also repeated the main analysis using a weighted risk score. We assigned a score value for each risk factor according to the range of FRs estimated in the multivariable model^29^ compared with fecundability (FR>0.8 = score 1; FR 0.7-0.8 = score 2; FR<0.7 = score 3).

To reduce the possibility of including individuals with subfertility in the analysis, we performed sensitivity analyses by excluding those who were attempting conception for more than 3 months (n = 389), 6 months (n = 258), and 12 months (n = 136) at study entry. We also excluded individuals with self-reported polycystic ovarian syndrome (n = 10). To examine whether the association was driven by maternal age, as it is known to be a factor in fecundability, we excluded maternal age from the risk score computation while adjusting for it in the model.

Assuming an unbiased association between the risk score and fecundability, we estimated the percentage reduction in the incidence of nonconception based on the population attributable fraction (if all participants achieved a minimum risk score level 1 [score of 0 or 1]). Data were presented as proportions of nonconception and 95% CIs. Significance was set at 2-sided *P* < .05. All analyses were performed using Stata, release 16 (StataCorp LLC), from March to May 2022.

## Results

**Figure 1.**
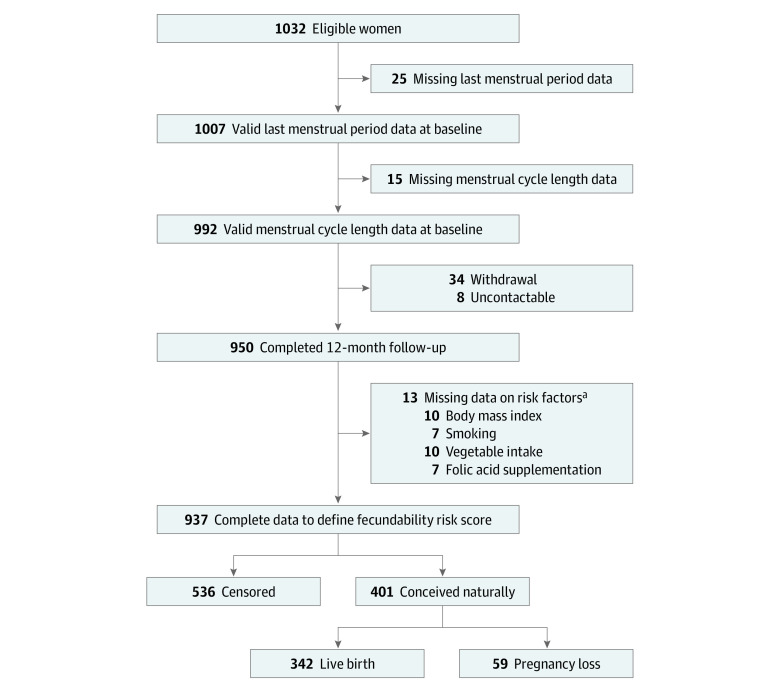
Flowchart of Study Participants ^a^Some participants had missing data on multiple risk factors.

Baseline sociodemographic characteristics, lifestyle factors, and attempted time to conception at study entry were similar among female patients who were included (n = 937) vs excluded (n = 95) (eTable 1 in Supplement 1). Of the included participants (mean [SD] age, 30.8 [3.8] years), 401 (42.8%) conceived spontaneously by contributing 3344 menstrual cycles over 1 year of follow-up, with 342 (36.5%) delivering live births. The median (IQR) number of cycles before conception was 4 (2-7). The remaining 536 patients (57.2%) were censored due to initiation of fertility treatment (n = 14), loss to follow-up (n = 19), or nonconception 1 year after recruitment (n = 503) (Figure 1).

Censored patients were more likely to be 32 years or older (253 [47.2%] vs 127 [31.7%]; *P* < .05); to have less than tertiary educational level (239 [44.6%] vs 109 [27.2%]; *P* < .05); to have lower monthly household income (79 [14.7%] vs 37 [9.2%]; *P* < .05); to have an unhealthy BMI (323 [60.3%] vs 182 [45.4%]; *P* < .05); to consume less than 2 servings per day of vegetables (396 [73.9%] vs 270 [67.3%]; *P* < .05); to achieve risk score level 5 (20 [3.7%] vs 3 [0.7%]; *P* < .05); and to have tried conceiving for more than 3 months (277 [51.7%] vs 112 [27.9%]; *P* < .05), 6 months (197 [36.8%] vs 61 [15.2%]; *P* < .05), and 12 months (115 [21.5%] vs 21 [5.2%]; *P* < .05) at study entry, compared with participants who successfully conceived (Table 1).

**Table 1.** Characteristics of Participants by Conception Status in the S-PRESTO Study

Characteristic	No. (%)		
	All participants (n = 937)	Participants who were censored (n = 536 [57.2%])^a^	Participants who conceived spontaneously (n = 401 [42.8%])
Age at recruitment: ≥32 y	380 (40.6)	253 (47.2)	127 (31.7)
Ethnicity			
Chinese	672 (71.7)	372 (69.4)	300 (74.8)
Indian	85 (9.1)	55 (10.3)	30 (7.5)
Malay	148 (15.8)	93 (17.4)	55 (13.7)
Mixed ethnicity^b^	32 (3.4)	16 (3.0)	16 (4.0)
Educational level:<tertiary level	348 (37.1)	239 (44.6)	109 (27.2)
Monthly household income, SGD			
Low: 1st-3rd decile	116 (12.4)	79 (14.7)	37 (9.2)
Middle: 4th-7th decile	625 (66.7)	351 (65.5)	274 (68.3)
High: 8th-10th decile	196 (20.9)	106 (19.8)	90 (22.4)
Parity: ≥1	323 (34.5)	173 (32.3)	150 (37.4)
Smoking status: ever smoker	96 (10.2)	63 (11.8)	33 (8.2)
Alcohol intake: >1 drink per wk	99 (10.6)	62 (11.6)	37 (9.2)
BMI: <18.5 or ≥23	505 (53.9)	323 (60.3)	182 (45.4)
Vegetable intake: <2 servings per d	666 (71.1)	396 (73.9)	270 (67.3)
Folic acid supplement nonuser	456 (48.7)	268 (50.0)	188 (46.9)
Risk score level			
Level 1: score of 0 or 1	209 (22.3)	88 (16.4)	121 (30.2)
Level 2: score of 2	318 (33.9)	179 (33.4)	139 (34.7)
Level 3: score of 3	277 (29.6)	173 (32.3)	104 (25.9)
Level 4: score of 4	110 (11.7)	76 (14.2)	34 (8.5)
Level 5: score of 5 or 6	23 (2.5)	20 (3.7)	3 (0.7)
Attempted time to conception at study entry, mo			
>3	389 (41.5)	277 (51.7)	112 (27.9)
>6	258 (27.5)	197 (36.8)	61 (15.2)
>12	136 (14.5)	115 (21.5)	21 (5.2)

Abbreviations: BMI, body mass index (calculated as weight in kilograms divided by height in meters squared); SGD, Singapore dollar; S-PRESTO, Singapore Preconception Study of Long-Term Maternal and Child Outcomes.

SI conversion factor: To convert SGD to US dollars, multiply by 0.74.

^a^ Included participants who initiated fertility treatment, were lost to follow-up, or did not get pregnant 1 year after recruitment.

^b^ Mixed ethnicity may include Chinese, Indian, or Malay ethnicity.

Across risk score levels, participants at higher level were more often of Malay ethnicity, had lower educational level, had lower monthly household income, were ever smokers, and were folic acid supplement nonusers. An increasing pattern of age, alcohol intake, and BMI and a decreasing pattern of vegetable intake were observed across the risk score levels (Table 2).

**Table 2.** Characteristics of Participants by Risk Score Level of Fecundability

Characteristic	Risk score level, No. (%)				
	Level 1: score of 0 or 1	Level 2: score of 2	Level 3: score of 3	Level 4: score of 4	Level 5: score of 5 or 6
No. of participants (%) (n = 937)	209 (22.3)	318 (33.9)	277 (29.6)	110 (11.7)	23 (2.5)
Age at recruitment, mean (SD), y	29.6 (2.9)	30.4 (3.4)	31.2 (4.4)	32.7 (3.4)	34.0 (2.8)
Ethnicity					
Chinese	168 (80.4)	240 (75.5)	176 (63.5)	73 (66.4)	15 (65.2)
Indian	17 (8.1)	24 (7.5)	30 (10.8)	12 (10.9)	2 (8.7)
Malay	20 (9.6)	41 (12.9)	59 (21.3)	23 (20.9)	5 (21.7)
Mixed ethnicity^a^	4 (1.9)	13 (4.1)	12 (4.3)	2 (1.8)	1 (4.3)
Educational level:<tertiary level	44 (21.1)	101 (31.8)	131 (47.3)	56 (50.9)	16 (69.6)
Monthly household income, SGD					
Low: 1st-3rd decile	15 (7.2)	30 (9.4)	41 (14.8)	26 (23.6)	4 (17.4)
Middle: 4th-7th decile	151 (72.2)	216 (67.9)	178 (64.3)	63 (57.3)	17 (73.9)
High: 8th-10th decile	43 (20.6)	72 (22.6)	58 (20.9)	21 (19.1)	2 (8.7)
Parity: ≥1	52 (24.9)	94 (29.6)	109 (39.4)	59 (54.1)	9 (39.1)
Smoking status: ever smoker	1 (0.5)	5 (1.6)	38 (13.7)	35 (31.8)	17 (73.9)
Alcohol intake, median (IQR), drinks per wk	0 (0-0.2)	0 (0-0.3)	0 (0-0.4)	0.1 (0-0.9)	0.8 (0-1.9)
BMI, mean (SD)	21.6 (3.2)	23.1 (4.9)	25.4 (5.8)	25.4 (6.0)	28.3 (4.6)
Vegetable intake, mean (SD), servings per d	2.2 (1.2)	1.7 (1.1)	1.5 (0.9)	1.2 (0.5)	1.2 (0.6)
Folic acid supplement nonuser	24 (11.5)	134 (42.1)	180 (65.0)	98 (89.1)	20 (87.0)
Attempted time to conception at study entry, median (IQR), menstrual cycles	1 (0-5)	1 (0-7)	1 (0-8)	1 (0-9)	5 (0-11)
Time to conception, median (IQR), menstrual cycles	4 (2-6)	4 (2-7)	5 (2-9)	6 (2-12)	NA

Abbreviations: BMI, body mass index (calculated as weight in kilograms divided by height in meters squared); NA, not applicable; SGD, Singapore dollar.

SI conversion factor: To convert SGD to US dollars, multiply by 0.74.

^a^ Mixed ethnicity may include Chinese, Indian, or Malay ethnicity.

**Figure 2.**
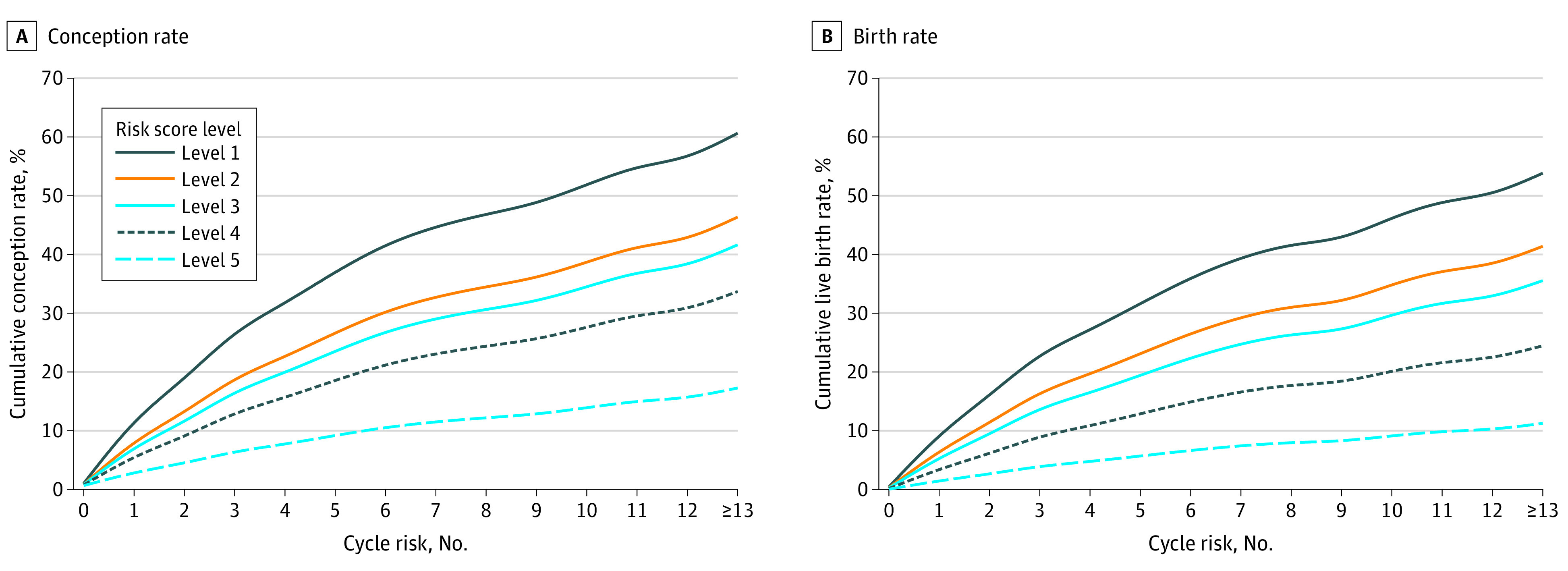
Cumulative Conception and Live Birth Rates by Risk Score Levels The cumulative percentages were estimated using the Kaplan-Meier method. The curves are adjusted for ethnicity, educational level, monthly household income, and parity.

Participants with a lower risk score level were more likely to conceive or deliver live births than those with a higher risk score level (Figure 2). Compared with participants with a level 1 risk score, those with level 2, 3, 4, and 5 risk scores had reductions in fecundability of 31% (FR, 0.69; 95% CI, 0.54-0.88), 41% (FR, 0.59; 95% CI, 0.45-0.78), 54% (FR, 0.46; 95% CI, 0.31-0.69), and 77% (FR, 0.23; 95% CI, 0.07-0.73), respectively (Table 3). For each additional level, fecundability was reduced by 23% (FR, 0.77; 95% CI, 0.69-0.85). Similar findings were observed when the event of interest was a conception resulting in a live birth (eTable 2 in Supplement 1). The square of the risk score variable, which appeared to be nonsignificant, demonstrated the absence of nonlinearity between risk score level and fecundability. If all participants had a level 1 risk score, the overall incidence of nonconception within a year would be reduced by 34% (95% CI, 30%-39%).

**Table 3.** Association Between Risk Score and Fecundability^a^

Risk score level	No. of participants (n = 937)	No. of pregnancies	No. of menstrual cycles	FR (95% CI)		PAF of nonconception at level 1, % (95% CI)^c^
				Crude model	Adjusted model^b^	
Level 1: score of 0 or 1	209	121	1048	1 [Reference]	1 [Reference]	34.3 (30.3-38.5)
Level 2: score of 2	318	139	1074	0.67 (0.53-0.86)	0.69 (0.54-0.88)	NA
Level 3: score of 3	277	104	884	0.56 (0.43-0.73)	0.59 (0.45-0.78)	NA
Level 4: score of 4	110	34	325	0.44 (0.30-0.65)	0.46 (0.31-0.69)	NA
Level 5: score of 5 or 6	23	3	13	0.20 (0.07-0.65)	0.23 (0.07-0.73)	NA
Per additional level	937	401	3344	0.75 (0.68-0.83)	0.77 (0.69-0.85)	NA

Abbreviations: FR, fecundability ratio; NA, not applicable; PAF, population attributable fraction.

^a^ Data were analyzed using discrete-time proportional hazards models.

^b^ Adjusted for ethnicity, educational level, monthly household income, and parity.

^c^ The proportion of nonconception that might have been reduced if all participants had achieved the minimum risk score level 1.

No substantial changes in FRs were observed when healthful PDI was used to replace vegetable intake as the diet component in the risk score (eTable 3 in Supplement 1). The use of a weighted risk score method revealed similar results to the equally weighted risk score (eTable 4 in Supplement 1). We observed similar findings when sensitivity analyses were performed, which restricted to participants with an attempted time to conception of 6 months or less (n = 679) or 12 months or less (n = 801) at study entry; the decrease in fecundability was greater when including only participants with an attempted time to conception of 3 months or less at study entry (n = 548) (eTable 5 in Supplement 1). Similar findings were observed when restricting to those without reported polycystic ovarian syndrome (n = 927) or when excluding maternal age from the risk score computation (eTable 6 in Supplement 1).

## Discussion

Using data from the S-PRESTO prospective cohort study of females of reproductive age who were trying to conceive, we developed a simple risk scoring system to examine the fecundability of these participants. The risk score comprised 6 modifiable factors associated with reduced fecundability: unhealthy BMI, unhealthy diet, nonuse of folic acid supplements, smoking, alcohol intake, and older age. A greater reduction in fecundability was observed in participants with a higher risk score level. Eliminating these risk factors to achieve a minimal risk level was estimated to reduce the incidence of nonconception by 34% in the study population.

Our choice of these modifiable risk factors was based on their association with fecundability in the literature. Both underweight and overweight or obesity are well-known risk factors for reduced fecundability.^30,31,32^ Lower adherence to healthful PDI was associated with lower fecundability.^12^ However, PDI calculation is complex and requires detailed dietary information, which limits its ease of use. Therefore, we used the vegetable component as a surrogate for a healthful plant-based dietary pattern. Natural antioxidants and flavonoids that are widely prevalent in vegetables may play a role in the control or reduction of the adverse outcome of oxidative stress for reproductive function.^33^ In this study, we did not include passive smokers given that active smoking is known to be associated with reduced fecundability.^20,21^ Similarly, alcohol intake and nonuse of folic acid supplements have been consistently associated with reduced fecundability.^14,15,16,17,34,35,36,37^ Other micronutrient supplements were not included in the risk score due to the lack of evidence on their role in fecundability. Although temporally nonmodifiable, planning to conceive at a younger age is an important modifiable decision, with both natural fertility and fertility treatment success declining with age.^1,2^ Inclusion of age in the screening tool supports raising awareness about the risks of delaying parenthood and facilitates informed family planning by individuals or couples. We did not include caffeine intake because its association with fecundability has not been conclusively established.^28,36,38^ Similarly, physical activity was not included due to the uncertain and mixed findings in the literature, including an inverse association^39^ between vigorous activity and fecundability and absent or weak positive association^40,41^ between moderate activity and fecundability.

Current practice in fertility management is reactive, wherein individuals or couples seek medical attention only when they are unable to conceive. We advocate for a more proactive approach that includes risk-based screening and early education to mitigate subfertility. For those with no immediate intention to become pregnant, the reduced fecundability risk scoring system serves as a functional public health tool that permits self-evaluation and provides guidance for early intervention to optimize reproductive health and family planning.^42^ When individuals or couples are ready to start a family, clinicians can use this tool to stratify patients according to their risk groups, counsel them on these modifiable risk factors, and take targeted steps to decrease their risk of nonconception. Early preventive and anticipatory guidance may be the key to addressing the subfertility epidemic and improving global fertility rates.

### Strengths and Limitations

The main strength of this study is the prospective cohort design of the S-PRESTO study, which recruited females before conception from the community, limiting recall bias in data collection. We designed a novel and practical modifiable risk factor scoring system, which has the potential to estimate fecundability in individuals attempting to conceive. Since the use of the weighted risk score method revealed results that were similar to those of an equally weighted risk score, a simple scoring system of assigning a single point to every risk factor makes this scoring system a user-friendly self-administered tool. The association between a higher risk score level and a lower live birth rate suggests the relevance and potential use of this risk scoring assessment tool in those planning to conceive.

We also recognize the limitations of this study. First, a longitudinal measure of lifestyle practices, which may change over time and affect fecundability, was not performed. Second, the associations and public health implications were specific to the study population, given that the conception rate in this study was 42.8%, which is lower than the global fertility rate of 80% to 90%.^43^ Third, this study involved females who mostly had higher educational levels, which may be associated with healthier lifestyle practices in the present sample.^44^ This variable might underestimate the population attributable fraction. Nevertheless, the low conception rate observed in this study is consistent with the low total fertility rate in Singapore and is similar to the conception rate in China.^45^ Fourth, paternal factors (eg, smoking and drinking) and sexual history (eg, intercourse frequency) were not collected in the study, limiting the comprehensiveness of the tool. Fifth, the cutoff values used for each risk factor may not be generalizable to other populations. For example, the lower BMI threshold (<23) is not applicable to those of White race and ethnicity. Future studies should explore the fecundability outcomes of different risk scoring models that are derived from various cutoff points for the included risk factors. Sixth, the small sample size, especially at the low- and high-risk scores, may limit the performance of this tool.

## Conclusions

In this cohort study, we proposed a novel risk scoring tool for fecundability that was based on 6 modifiable risk factors, including unhealthy BMI, unhealthy diet, smoking, alcohol intake, nonuse of folic acid supplements, and older maternal age. A greater reduction in fecundability was observed in participants with a higher risk score level. Achieving a risk score of 0 or 1 by all participants was estimated to reduce the incidence of nonconception by 34% in the study population. This tool can be self-administered to empower individuals or couples to mitigate their risks as they plan for their families and to guide health care practitioners in making recommendations for those who are trying to conceive. However, future studies are needed to externally validate this tool in different populations globally and to evaluate its generalizability and performance in both public health and clinical practice.

## Notes

Supplement 1. **eTable 1.** Characteristics of Participants According to Their Inclusion Status in the Present Study **eTable 2.** Association Between Risk Score and Fecundability, Based on Singleton Live Birth as Outcome Measure **eTable 3.** Association Between Risk Score and Fecundability, Using Healthful Plant-Based Diet Index at Score of ≤47 to Indicate Unhealthy Diet in the Risk Score **eTable 4.** Association Between Weighted Risk Score and Fecundability **eTable 5.** Association Between Risk Score and Fecundability Among Women With Conception Attempts of ≤3, ≤6 and ≤12 Months at Study Entry **eTable 6.** Association Between Risk Score and Fecundability When Excluding Women With Polycystic Ovarian Syndrome (PCOS) Reported or When Excluding Maternal Age Factor From the Risk Score

Supplement 2.
Data Sharing Statement
