# Supplementary material for: Modifiable Risk Factor Score and Fecundability in a Preconception Cohort in Singapore
Source: JAMA Netw Open. 2023 Feb 7;6(2):e2255001. doi: 10.1001/jamanetworkopen.2022.55001 (PMC10408273; doi:10.1001/jamanetworkopen.2022.55001)
Supplement: Supplement 1. — eTable 1. Characteristics of Participants According to Their Inclusion Status in the Present Study eTable 2. Association Between Risk Score and Fecundability, Based on Singleton Live Birth as Outcome Measure eTable 3. Association Between Risk Score and Fecundability, Using Healthful Plant-Based Diet Index at Score of ≤47 to Indicate Unhealthy Diet in the Risk Score eTable 4. Association Between Weighted Risk Score and Fecundability eTable 5. Association Between Risk Score and Fecundability Among Women With Conception Attempts of ≤3, ≤6 and ≤12 Months at Study Entry eTable 6. Association Between Risk Score and Fecundability When Excluding Women With Polycystic Ovarian Syndrome (PCOS) Reported or When Excluding Maternal Age Factor From the Risk Score [file jamanetwopen-e2255001-s001.pdf]

Supplemental Online Content

Loy SL, Ku CW, Tiong MMY, et al. Modifiable risk factor score and fecundability in a preconception cohort in Singapore. *JAMA Netw Open*. 2023;6(2):e2255001. doi:10.1001/jamanetworkopen.2022.55001

eTable 1. Characteristics of Participants According to Their Inclusion Status in the Present Study

eTable 2. Association Between Risk Score and Fecundability, Based on Singleton Live Birth as Outcome Measure

eTable 3. Association Between Risk Score and Fecundability, Using Healthful Plant-Based Diet Index at Score of ≤47 to Indicate Unhealthy Diet in the Risk Score

eTable 4. Association Between Weighted Risk Score and Fecundability

eTable 5. Association Between Risk Score and Fecundability Among Women With Conception Attempts of ≤3, ≤6 and ≤12 Months at Study Entry

eTable 6. Association Between Risk Score and Fecundability When Excluding Women With Polycystic Ovarian Syndrome (PCOS) Reported or When Excluding Maternal Age Factor From the Risk Score

This supplementary material has been provided by the authors to give readers additional information about their work.

© 2023 Loy SL et al. *JAMA Network Open.*

eTable 1. Characteristics of participants according to their inclusion status in the present study

	No. (%)		
**Characteristics**	**Included (n=937)**	**Excluded (n=95)**[a]	**p**[b]
Age at recruitment ≥32 years	380 (40.6)	35 (36.8)	0.482
Ethnicity			0.725
Chinese	672 (71.7)	71 (74.7)	
Indian	85 (9.1)	10 (10.5)	
Malay	148 (15.8)	11 (11.6)	
Mixed ethnicity[c]	32 (3.4)	3 (3.2)	
Educational level: <tertiary level	348 (37.1)	36 (43.9)	0.226
Monthly household income (SGD)			0.762
Low: 1st - 3rd decile	116 (12.4)	14 (14.7)	
Middle: 4th - 7th decile	625 (66.7)	63 (66.3)	
High: 8th - 10th decile	196 (20.9)	18 (18.9)	
Parity ≥1	323 (34.5)	31 (41.9)	0.200
Smoking status: ever smoker	96 (10.2)	11 (14.9)	0.214
Alcohol intake >1 drink per wk	99 (10.6)	12 (12.6)	0.536
BMI: <18.5 or ≥23	505 (53.9)	39 (55.7)	0.768
Vegetable intake: <2 servings per d	666 (71.1)	48 (68.6)	0.656
Folic acid supplement nonuser	456 (48.7)	34 (45.9)	0.652
Risk score level			0.115
Level 1: score of 0 or 1	209 (22.3)	18 (27.3)	
Level 2: score of 2	318 (33.9)	16 (24.2)	
Level 3: score of 3	277 (29.6)	17 (25.8)	
Level 4: score of 4	110 (11.7)	14 (21.2)	
Level 5: score of 5 or 6	23 (2.5)	1 (1.5)	
Attempted time to conception at study entry, mo			
>3	389 (41.5)	31 (32.6)	0.093
>6	258 (27.5)	24 (25.3)	0.636
>12	136 (14.5)	12 (12.6)	0.618

Abbreviations: BMI, body mass index (calculated as weight in kilograms divided by height in meters squared); SGD, Singapore dollar
SI conversion factor: To convert SGD to US dollars, multiply by 0.74.
[a]Sample size of excluded women does not always equal to 95 due to missing values.
[b]Based on Pearson's chi-squared test.
[c]Mixed ethnicity may include Chinese, Indian, or Malay ethnicity.

© 2023 Loy SL et al. *JAMA Network Open.*

eTable 2. Association between risk score and fecundability, based on singleton live birth as outcome measure[a]

	Live births	Crude model	Adjusted model[b]
**Risk score**	**n**	**FR (95% CI)**	**FR (95% CI)**
Level 1: score of 0 or 1	104	1.00 [Reference]	1.00 [Reference]
Level 2: score of 2	124	0.70 (0.54, 0.91)	0.72 (0.55, 0.93)
Level 3: score of 3	88	0.55 (0.42, 0.73)	0.59 (0.44, 0.78)
Level 4: score of 4	24	0.36 (0.23, 0.57)	0.39 (0.24, 0.61)
Level 5: score of 5 or 6	2	0.16 (0.04, 0.66)	0.18 (0.04, 0.73)
Per additional level	342	0.65 (0.58, 0.72)	0.74 (0.65, 0.83)

Abbreviations: FR, fecundability ratio; CI, confidence interval.
[a]Data were analyzed using discrete-time proportional hazards models.
[b]Adjusted for ethnicity, educational level, monthly household income, and parity.

© 2023 Loy SL et al. *JAMA Network Open.*

eTable 3. Association between risk score and fecundability, using healthful plant-based diet index at score of ≤47 to indicate unhealthy diet in the risk score[a]

	Crude model	Adjusted model[b]
**Risk score**	**FR (95% CI)**	**FR (95% CI)**
Level 1: score of 0 or 1	1.00 [Reference]	1.00 [Reference]
Level 2: score of 2	0.60 (0.48, 0.77)	0.61 (0.48, 0.78)
Level 3: score of 3	0.64 (0.50, 0.83)	0.69 (0.53, 0.90)
Level 4: score of 4	0.40 (0.27, 0.60)	0.42 (0.28, 0.63)
Level 5: score of 5 or 6	0.13 (0.02, 0.90)	0.13 (0.02, 0.95)
Per additional level	0.76 (0.68, 0.85)	0.77 (0.69, 0.85)

Abbreviations: FR, fecundability ratio; CI, confidence interval.
[a]Data were analyzed using discrete-time proportional hazards models.
[b]Adjusted for ethnicity, educational level, monthly household income, and parity.

© 2023 Loy SL et al. *JAMA Network Open.*

eTable 4. Association between weighted risk score and fecundability[a]

	Crude model	Adjusted model[b]
**Weighted risk score[c]**	**FR (95% CI)**	**FR (95% CI)**
Level 1: score of 0 or 1	1.00 [Reference]	1.00 [Reference]
Level 2: score of 2 or 3	0.59 (0.41, 0.84)	0.59 (0.42, 0.83)
Level 3: score of 4 or 5	0.47 (0.33, 0.66)	0.49 (0.35, 0.69)
Level 4: score of 6 or 7	0.37 (0.25, 0.56)	0.38 (0.25, 0.56)
Level 5: score of 8 or 10	0.22 (0.13, 0.39)	0.22 (0.13, 0.39)
Per additional level	0.73 (0.70, 0.78)	0.85 (0.81, 0.90)

Abbreviations: FR, fecundability ratio; CI, confidence interval.
[a]Data were analyzed using discrete-time proportional hazards models.
[b]Adjusted for ethnicity, educational level, monthly household income, and parity.
[c]The weighted risk score values were estimated based on the range of FRs for each risk factor in the multivariable discrete-time proportional hazards model in relation to fecundability. Score 1: >FR 0.8; Score 2: FR 0.7-0.8; Score 3: FR <0.7.

© 2023 Loy SL et al. *JAMA Network Open.*

eTable 5. Association between risk score and fecundability among women with conception attempts of ≤3, ≤6 and ≤12 months at study entry[a]

	≤3 months conception attempts before study entry (n=548)	≤6 months conception attempts before study entry (n=679)	≤12 months conception attempts before study entry (n=801)
**Risk score**	**FR (95% CI)**	**FR (95% CI)**	**FR (95% CI)**
Level 1: score of 0 or 1	1.00 [Reference]	1.00 [Reference]	1.00 [Reference]
Level 2: score of 2	0.57 (0.34, 0.96)	0.71 (0.54, 0.93)	0.75 (0.58, 0.96)
Level 3: score of 3	0.33 (0.18, 0.61)	0.58 (0.43, 0.77)	0.63 (0.48, 0.84)
Level 4: score of 4	0.29 (0.13, 0.65)	0.49 (0.32, 0.75)	0.51 (0.34, 0.76)
Level 5: score of 5 or 6	0.35 (0.06, 1.86)	0.36 (0.11, 1.13)	0.27 (0.08, 0.84)
Per additional level	0.65 (0.52, 0.82)	0.78 (0.69, 0.87)	0.79 (0.71, 0.88)

Abbreviations: FR, fecundability ratio; CI, confidence interval.
[a]Data were analyzed using discrete-time proportional hazards models, adjusting for ethnicity, educational level, monthly household income, and parity.

© 2023 Loy SL et al. *JAMA Network Open.*

eTable 6. Association between risk score and fecundability when excluding women with polycystic ovarian syndrome (PCOS) reported or when excluding maternal age from the risk score[a]

	Excluding PCOS cases			Excluding maternal age factor
Risk score	FR (95% CI)[b]		Risk score	FR (95% CI)[c]
Level 1: score of 0 or 1	1.00 [Reference]		Level 1: score of 0 or 1	1.00 [Reference]
Level 2: score of 2	0.67 (0.54, 0.85)		Level 2: score of 2	0.73 (0.57, 0.93)
Level 3: score of 3	0.58 (0.45, 0.75)		Level 3: score of 3	0.67 (0.50, 0.89)
Level 4: score of 4	0.49 (0.34, 0.71)		Level 4: score of 4 or 5	0.51 (0.34, 0.76)
Level 5: score of 5 or 6	0.23 (0.07, 0.70)			
Per additional level	0.77 (0.69, 0.86)		Per additional level	0.81 (0.72, 0.91)

Abbreviations: FR, fecundability ratio; CI, confidence interval.
[a]Data were analyzed using discrete-time proportional hazards models.
[b]Adjusted for ethnicity, educational level, monthly household income, and parity.
[c]Adjusted for ethnicity, educational level, monthly household income, parity, and maternal age.

© 2023 Loy SL et al. *JAMA Network Open.*
