# Supplementary material for: Modifiable Risk Factor Score and Fecundability in a Preconception Cohort in Singapore
Source: JAMA Netw Open. 2023 Feb 7;6(2):e2255001. doi: 10.1001/jamanetworkopen.2022.55001 (PMC10408273; doi:10.1001/jamanetworkopen.2022.55001)
Supplement: Supplement 2. — Data Sharing Statement [file jamanetwopen-e2255001-s002.pdf]

Data Sharing Statement

Loy. Modifiable Risk Factor Score and Fecundability in a Preconception Cohort in Singapore. *JAMA Netw Open*. Published February 07, 2023. doi:10.1001/jamanetworkopen.2022.55001

Data
Data available: No

Additional Information
Explanation for why data not available: Restrictions apply to the availability of these data, which were used under license for this study. Data are available upon request and approval by the S-PRESTO committee.
